# A protocol for developing a complex needs indicator for veterans (CNIV) in the UK

**DOI:** 10.1016/j.puhip.2022.100281

**Published:** 2022-06-11

**Authors:** Anastasia Fadeeva, Ajay Tiwari, Emily Mann, Matthew D. Kiernan

**Affiliations:** a Faculty of Health and Life Sciences, Northumbria University, Newcastle upon Tyne, United Kingdom; b Centrum Wiskunde & Informatica, Amsterdam, Netherlands; c Violence and Society Centre, City, University of London, London, UK

**Keywords:** Veterans, Complex needs, Charities, Health and social care, Composite indicators

## Abstract

**Introduction:**

The veteran population in the UK has been decreasing, however, there remains a proportion of veterans and their families who continue to experience multiple and complex health, financial, and social needs. The complex problems tend to exacerbate each other and deepen over time if appropriate support is not provided. Identifying the veterans with complex needs is crucial for effective support by military charities and health and social care services. The present research aims to develop a complex needs indicator for the veteran population (CNIV) that will quantify complexity and help to identify the risk of having or developing complex needs.

**Methods:**

The development of the CNIV will be informed by the guidance for constructing composite indicators. The data on grant support received by veterans’ beneficiaries from the UK Royal Marine and SSFA charities will be used for designing the indicator and evaluating its robustness. The crucial step in constructing the indicator is assigning weights to different needs and risk factors associated with complex cases. Factor analysis (FA) and analytical network process (ANP) will be used as weighting methods for the analysed variables.

**Conclusion:**

The development of CNIV has important implications for research and practice, such as the potential to be used as a screening tool for identifying complex cases, improved provision of the targeted support to veterans, assessing the scope of complex problems among veterans within the country and informing policy makers and a more general audience of the complexity of need within the sector.

## 1 Introduction

### 1.1 Complex needs of veterans

Following the end of National Service in the UK in 1957, the size of the British Armed forces has contracted year on year following multiple defence reviews, for example between 2010 and 2015 the UK Army was reduced from 102,000 trade-trained personnel to 82,000 and is set to reduce to 72,500 by 2025 [1]. Subsequently, the size of the veteran community has contracted accordingly. As the National Service veterans near end of life, it is expected that the size of the UK veteran population will shrink significantly in the coming years from 2.5 million in 2016 to 1.6 million by 2028 [2]. However, following the wars in Afghanistan and Iraq, the advances in trauma care and survivability, and an ever-increasing expectation of support by individuals in wider society, it is argued that whilst the veteran population may be decreasing, the complexity of needs and the support required by individuals is increasing year on year [3]. Whilst many UK service leavers make a successful transition to civilian life, there is still a significant number of veterans who experience complex physical, mental, and social problems [4]. Studies examining the health challenges experienced by veterans demonstrate complex mental health issues [4], substance misuse [5], and physical health conditions and disability [6]. Beyond health, other studies highlight that veterans experience issues with housing or the ability to establish a safe living environment [7], the risk of social isolation [8], as well as the risk of experiencing financial hardship [9]. These needs can be complex and concurrent, for example, it is not uncommon that a mental health issue such as PTSD may combine with physical illnesses, substance misuse, financial and/or social challenges [[10], [11], [12]].

Complex problems require more comprehensive interventions such as case management support as well as significant time, financial, and human resources [13]. Those with complex needs are usually hardest to reach and more difficult to support, which in turn increases the risk of exacerbating the individual's multiple problems causing significant human cost [14]. Therefore, to better support the veteran population, it is crucial to understand which individuals might be at higher risk of having complex needs. Moreover, a recent report published by the All Party Parliamentary Group (APPG) on Complex Needs and Dual Diagnosis highlighted the need for further evidence to understand the complex needs faced by different groups within the UK [15]. Additionally, without understanding the complexity of beneficiaries needs it is challenging to estimate whether the provision of support is sufficient. Therefore, the Ministry of Defence and armed force charities emphasised the need for more work to improve veteran beneficiaries need assessment [16].

The identification of complex needs can be a challenging task, as it requires a complex assessment of individual circumstances and needs, gathering comprehensive information from the individual and extensive communication between services, and therefore would require considerable resources. Veterans' charities manage numerous cases for support of veterans and their families, but, generally, do not employ methods to differentiate between cases in terms of their complexity. This process might be facilitated through employing a screening method for the immediate assessment of individual cases and complexity of veterans' needs. Such a tool could alert practitioners, service providers and charities about individuals with potentially complex needs and encourage to explore the breadth and depth of the cases where necessary. Additionally, the proposed method can be used by research and charity sector to estimate the trends on changes in complexity of veterans’ need over time and understand whether cases have become more complex recently.

An assessment tool with a potential to be employed in research and wider practice could be a scoring system that helps to assess the risk of developing or having complex needs for each veteran. Given that complex needs assume multiple needs that interact and exacerbate each other [15], the score for each individual can be calculated based on the number of needs the individual has and the risk of each problem being associated with a complex case.

### 1.2 Composite indicators

Due to the increasing amount of data and metrics, there has been a growing demand for methods that could assist with interpreting complex problems and consolidating multidimensional information in different areas of research, policy, and practice. This demand has led to an increasing interest in and use of composite indicators, to better understand various phenomena [17]. According to the OECD's definition, a composite indicator “is formed when individual indicators are compiled into a single index, on the basis of an underlying model of the multi-dimensional concept that is being measured”. Composite indicators are used to summarise information about complex cases in a single number, which makes it easier to understand, compare, and rank different cases [17,18]. However, development of composite indicators is a comprehensive process that typically requires a number of decisions to be made. The literature [18,19] has described numerous steps in the construction of composite indicators from developing a theoretical framework to presenting the results.

One of the most crucial steps in constructing composite indicators is weighting, which involves assigning weights to variables contributing to the main indicator. Selecting a weighting method can be a challenging task. A primary classification of weighting methods distinguishes equal weighting, which implies that the same weights are assigned to all sub indicators, and unequal weighting, which assigns different weights to the variables or sub indicators based on some prior knowledge about their relative importance [17]. Methods for estimating unequal weights are further divided into data driven (“objective”) that use statistical analyses and knowledge driven (or “subjective”) approaches that rely on experts’ consensus regarding the importance of sub indicators [18]. However, the range of weighting methods described in the literature has numerous limitations. In particular, “subjective” methods can be prone to errors of judgement and biases, may produce inconsistent results, especially when the phenomena to be measured is not well defined, and/or experts need to deal with a large number of underlying sub indicators [17]. With regards to “objective” methods, one of the criticisms is that calculated relationships between sub indicators (e.g., correlations) do not always represent the influence between them [19].

Recently, there has been an increasing use in hybrid approaches that include a combination of both data driven and knowledge driven methods [20]. It is argued that hybrid approaches are the most suitable for developing composite indictors, since they combine various decision-making tools, include a series of possible alternatives for the analysis (i.e., ranking, comparing), and consider different criteria simultaneously. For example, Zebardast [21] combined factor analysis (FA) and analytic network process (ANP [22]) for weighting and aggregating vulnerability indicators into a composite index of social vulnerability to earthquake hazards. In the first step, the described method applied data-driven approach FA as an exploratory tool to extract different dimensions of social vulnerability and key variables associated with these dimensions. In the second step, ANP used the results of FA to calculate the relative weights of the variables. Not only can the method proposed by Zebardast [21] help handle the problem of interdependence among the variables, it relies on absolute measurements obtained through the FA in weighting instead of more “subjective” experts’ opinions.

### 1.3 Aim of the study

With regards to complex needs, to our knowledge, no prior research has attempted to assign weights to various financial and non-financial needs of the veterans' population and develop a scoring method for their complex needs. Given the need for a method that would help to identify veterans with complex needs, the present research will aim to develop a complex needs indicator for the veteran population (CNIV) that will quantify complexity and help to identify the risk of having or developing complex needs. We will be guided by the comprehensive recommendations for constructing and evaluating composite indicators described in the literature [17,18]. Due to the importance of accounting for the interaction between different needs and risk factors, the hybrid weighting method proposed by Zebardast [21] will be applied to develop the CNIV. We will use the dataset collected by the UK Royal Marine and SSAFA charity on their beneficiaries grant applications from 2014 to 2019. The calculated weights will be then used to estimate the changes in complexity of the beneficiaries’ needs during the period of data collection.

We hypothesise that the complexity of the veterans’ needs increased year on year between 2014 and 2019.

## 2 Methods

### 2.1 Study population

The UK Royal Marine and SSAFA benefit recipients between 2014 and 2019. All the included beneficiaries are UK veterans.

### 2.2 Data analysis

Step 1. Selecting variables

The initial stage of developing the CNIV followed the first steps described by Nardo, Saisana [18], which includes defining the phenomenon to be measured and selecting variables in accordance with theoretical knowledge. The APPG on Complex Needs and Dual Diagnosis distinguished the following categories of needs: physical and mental health, education/employment, poverty/financial hardship and food poverty, and housing [15]. These needs typically interact and exacerbate each other, which causes people to experience several issues simultaneously. As a result, the needs may become long-standing and require multiple types of support.

The APPG conceptualisation of need, with need being determined as a shortage in a certain life domain which requires some form of external support, reflects the definition suggested by Darcy and Hofmann [23] where need is defined as being ‘relief assistance or some other humanitarian intervention’. This conceptualised definition of need has informed the selection of variables for the analysis in the present research. For constructing the CNIV, we will use datasets from a UK military charity that support beneficiaries for various needs. The variables will be selected based on the definition of complex needs provided by the APPG and grant categories for different types of the beneficiaries needs, such as medical treatment and debt relief (see Table 1). The data on the number of times ttbl1he grants support was requested will be used as a value for each need, as it indicates the number of potential interventions required to address the problems and thus the potential depth of the issues.

Step 2. Missing data

**Table 1:** Variables selected for the CNIV development.

	Variable	Measure
**Need categories**	Care at home / Social Services	Number of times the corresponding support was provided.
	Debt/Financial	
	Counselling / Mental Health Services	
	Essential Clothing	
	Essential Food and Groceries	
	Household Goods	
	Housing	
	Medical	
	Mobility Support	
	Legal support	
	Training/Education	
	Children's needs	

If less than 5% of data is missing, listwise deletion will be applied [24]. If the missing data exceeds the recommended maximum, maximum likelihood multiple imputation (MLMI) will be used. Maximum likelihood is a relatively fast imputation method that produces consistent and accurate efficient point estimates [25].

Step 3. Normalisation

The selected variables will be transformed and standardised using Min-Max transformation (see Eq. (1)) before proceeding with the next steps of the analysis [21].

(1) $$TX_{i}=\frac{X_{i}-X_{iMin}}{X_{iMax}-X_{iMin}}$$

Where *TX*_*i*_ is the transformed value of the original variable *X*_*i*_, *X*_*iMax*_ and *X*_*iMin*_ are the maximum and minimum values of the original variable *X*_*i*_ respectively.

Step 4. Multicollinearity check

Before applying FA, multicollinearity check should be performed. The Kaiser-Meyer-Olkin (KMO), which is measure of sampling adequacy and the Bartlett's test of sphericity are recommended to check correlations between indicators [18]. The KMO should be above 0.5 to proceed with FA [26]. Additionally, Bartlett's test of sphericity, which tests the null hypothesis that the individual indicators in a correlation matrix are uncorrelated, will be performed.

Step 5. Factor analysis

In the next phase, FA will be applied to the identified variables to extract the underlying dimensions of the investigated phenomena and the key variables associated with these dimensions. The total number of factors to be extracted will be determined in accordance with the Kaiser [27] rule, that is only factors with eigenvalues greater than or equal to one are accepted as possible sources of variance in the data, and the greatest priority is ascribed to the factor that has the highest eigenvector sum. Rotation method will be selected to maximise loadings of individual variables on individual factors [18]. A variable will be assigned to a factor with the highest loading on that factor.

Step 6. Analytic Network Process

In the next step, ANP [22] will be used to construct a network model for the component loadings obtained from FA, to calculate the relative weights of complex needs indicators.

Analytic Network Process is a widely used procedure in multi-decision making, which decomposes the problem into a hierarchy consisting of three levels: the ultimate goal, the criteria, and the alternatives [28]. The core element of ANP is a pairwise comparison of its attributes. Instead of relying on expert judgments, which use subjective opinions to assign the importance to each criteria, Zebardast [21] suggested to use absolute measurements obtained through the FA - variance explained by each factor and loadings of indicators on each factor. Therefore, our study will apply the results of FA as a measure of importance for the obtained factors and variables in building pairwise comparison matrices (see Table 2).

Step 7. Aggregation

**Table 2:** Description of the ANP steps for obtaining relative weights of the CNIV variables.

Steps of ANP	Description
**Constructing the model and problem structuring**	Defining the overall objective of the study - that is building complex needs indicator, the criteria – the dimensions extracted by FA, and the attributes – primarily variables of the extracted factors.
**Constructing pairwise comparison matrix and priority vectors for the criteria.**	Amount of variance explained by each factor extracted in FA will be used as the importance measure in developing pairwise comparison matrix. After constructing corresponding comparison matrices, the local priority vectors for the factors will be computed. Priority vectors can be viewed as importance measures that have been transformed into relative form — that is, they have been normalised so that they sum to 1 [21].
**Constructing local priority matrix for interdependent variables.**	To determine the interdependency between the variables, a correlation analysis among the variables of each CNIV dimension will be conducted separately. The variables that are significantly related to each other (*p* = .01) will be considered interdependent. The absolute values of corelations coefficients for the interdependent variables will be used as the measures of importance in constructing the corresponding pairwise comparison matrices and obtaining local priority matrices.
**Calculating the limit super matrix**	The calculated priority matrices will be entered into a super matrix. The super matrix is a partitioned matrix with each segment showing the relationships between two components in a system. The super matrix will be raised to a power of an arbitrary large number to calculate the limit super matrix, the goal column of which will present the absolute values of weights of CNIV variables. The obtained weights will be normalised and for the convenience of processing can be magnified [4].

To compute complex needs scores for each individual, the obtained weights will be aggregated by using a linear method as illustrated in Eq [2].:

(2) $$CNIV_{i}=\overset{v}{\underset{v=1}{\sum }}W_{ANP_{v}}SVI_{iv}$$

where, *CNIV*_*ί*_ is the complex needs indicator for an individual *‘ί’, W*_*ANPv*_ is the weight of the *CNIV*_*ί*_ sub indicator or variable *‘ν’* obtained from the limited super matrix, *SVI*_*ίν*_ is the standardised value of the *CNIV*_*ί*_ sub indicator *‘ν’* for an individual *‘ί’*.

Step 8. Robustness evaluation

To ensure the quality of the model and its assumptions, the evaluation of the modelling process and implemented steps will be conducted in the last step of constructing the CNIV.

The uncertainty of the model can be quantified by calculating the confidence intervals for the CNIVs obtained. The bootstrapping method will be utilised to calculate the confidence intervals for the CNIVs. Bootstrapping can be used to estimate the confidence intervals of uncertainty eliminating the need to do further sampling, even for a limited number of samples [29]. The bias-corrected and accelerated (BCa) bootstrap interval is utilised to calculate the confidence interval.

Step 9. Hypothesis testing

We hypothesise that the complexity of the veterans’ needs increased year on year between 2014 and 2019.

The estimated weights will be applied to calculate a mean and median values of the CNIV for each year (from 2014 to 2019). The hypothesis will be tested using generalised linear model with complex needs score CNIV being entered as a dependent variable. The time (measured in years) when grant application(s) from each beneficiary were made will be entered as a continuous predictor variable.

### 2.3 Study materials and instruments

Anonymised datasets of grant support received by veterans' beneficiaries from the Royal Marine and SSAFA charities. All the calculations and analysis will be done using MS Excel, R (version 4.1.0; packages ‘dplyr’, ‘psych’, ‘compind’, ‘ahpsurvey’, ‘boot’) and the Super Decisions software (www.superdecisions.com).

## 3 Conclusions

If the complexity of needs among UK veterans is increasing despite the shrinking veteran population, it might indicate a need for their better support. To inform government policy and determine the trend of case complexity, a reliable measure is required. In addition, prior research on veterans' needs in the UK highlight that those individuals with multiple needs are often less likely to seek or access support [[4], [5], [6],^8^]. The first step of providing better services for veterans is identifying individuals at higher risk of having or developing complex needs. Therefore, the aim of this study is to develop a complex needs indicator for veterans that can determine the size and prevalence of case complexity in the UK veteran's population and be used as a tool to screen individuals at greater risk of complex needs.

Complex needs indicator will incorporate the breadth of complex cases (i.e., number of different needs) and the depth of problems (i.e., the number of interventions required to address the problems over time). The important function of the proposed scoring method is also to account for the interdependencies between different needs. Therefore, the development of the CNIV will follow the steps for constructing composite indicators and use FA and ANP as weighting methods for the analysed variables.

The work undertaken by Vogt et al. (2018) seeks to develop a tool to determine wellbeing in a veteran population, yet no previous research has developed a weighting system to determine the complexity of needs of those veterans that seek help. More importantly, the calculation of CNIV will only require service usage information that has been requested and provided by organisations and charities that serve the armed forces sector. The advantage of the proposed method is that it will not require additional resources to recruit veteran participants and conduct the assessment and can be widely used by charities, for example for initial screening all their beneficiaries for complex needs. To develop a more comprehensive understanding of how complex needs affect people's functioning and well-being, the proposed assessment could be combined with self-report assessments of veterans' well-being, such as those developed by Vogt et al. [30].

The development of the CNIV has important implications. The index will assist in developing government policy and will help charity organisations, social and health care services, and researchers develop a better understanding of complex cases and help to improve the provision of support. It will allow synthetising the multi facet information about complex needs and presenting the results to the policy makers and general public in an effective manner. Furthermore, the CNIV can be used to assess the scope of distribution of complex needs among veterans, estimate changes in complexity over time and locate the geographic regions with more complex cases are, being an essential tool in determining the distribution of funding and resources. The construction of composite indicators requires large sequence of analytical steps and decisions. Therefore, we present our protocol for developing the CNIV for peer review to ensure the scientific rigour of the proposed methods.

## Funding

This project was funded by the Armed Forces Covenant Fund Trust, grant number: CF-SG001.

## Patient consent for publication

Not required.

## Ethics statement

The study was subject to the ethical review and has received ethical approval from the Anonymised University Ethics committee. Submission Ref: 768. All the Data will be completely anonymised before any analysis is undertaken.

## Data availability statement

Data cannot be shared publicly because the data belongs to third parties, the Royal Marines Charity and SSAFA, and shared under a legal data sharing agreement for the purpose of this study. In addition, the data contains sensitive information on the location of vulnerable veterans. Data from the study are available upon request from Anonymised for researchers who meet the criteria for access to confidential data and upon agreement of the Royal Marines Charity and SSAFA.

## Declaration of competing interest

The authors declare that they have no known competing financial interests or personal relationships that could have appeared to influence the work reported in this paper.
